# Neuromorphic engineering: Artificial brains for artificial intelligence

**DOI:** 10.1111/nyas.15256

**Published:** 2024-11-20

**Authors:** Johannes Leugering

**Affiliations:** ^1^ Institute for Neural Computation University of California San Diego La Jolla California USA

**Keywords:** AI, architecture, computation, neuromorphic engineering

## Abstract

Neuromorphic engineering is a research discipline that tries to bridge the gaps between neuroscience and engineering, cognition and algorithms, and natural and artificial intelligence. Neuromorphic engineering promises revolutionary breakthroughs that could rapidly advance our understanding of the brain and pave the way toward more human‐like and sustainable artificial intelligence. But first, it will have to find its way out of the laboratory.

## THE COMPUTER AND THE BRAIN

In 1943, Warren McCulloch and Walter Pitts^1^ proposed a radical idea: that biological neurons, much like electronic circuits, implement a universal logic calculus. Their proposed model was exceedingly simple: a neuron fires a *spike*, that is, it outputs “1,” whenever it receives enough stimulation; otherwise, it does not fire, outputting “0” instead. In sufficient numbers and with the right interconnections, such simple neurons could solve any problem that a Turing machine could, and *vice versa*.

This mechanistic view offered two exciting prospects: On the one hand, this meant that artificial neural networks could be *simulated* on computers, making it easier to study their behavior, and opening a path toward artificial intelligence (AI). Turing himself championed this idea,^2^ which has culminated in deep learning today.

On the other hand, this concept offered a blueprint for a fundamentally different kind of computer. Instead of relying on a central processor that executes a program, it would use artificial neurons as basic building blocks, and their behavior would be governed by the synaptic connections between them.

This alternative approach intrigued even John von Neumann, the architect of conventional computers, so much that he made it the topic of his final, unfinished book *The Computer and the Brain*.^3^ A digital computer, he observed, comprises very fast, precise, and inherently error‐correcting logic gates. This allows it to execute long sequences of (simple) operations without accumulating errors—a prerequisite for complex computer algorithms.

Biological neurons, however, are comparatively slow and imprecise, firing only around once per second. To solve hard problems quickly and reliably, the brain (or an artificial analog) must instead rely on a large number of neurons that operate in parallel and provide sufficient redundancy or adaptation to overcome the noise. Yet, in principle, nothing stands in the way of building artificial semiconductor circuits along the same lines. This insight is the foundation of *neuromorphic engineering—*although the term was not coined until much later. Early pioneers like Frank Rosenblatt set out to demonstrate this in practice.

Rosenblatt developed the perceptron,^4^ a neural network capable of learning to recognize 10 digits from rudimentary visual inputs. He first implemented it as a simulation running on an IBM mainframe, then he built a hardware version in which thousands of electric motors controlled potentiometers that set node connection strengths. Rosenblatt's machine made the news,^5^ causing some to speculate that human‐like artificial intelligence was just around the corner.

But because of limitations at the time of the learning algorithms, network topologies, and the hardware itself, such early attempts ultimately failed to scale to real‐world problems, and any initial excitement soon faded.

## RULED BY MOORE'S LAW

A decade later, when the first integrated microprocessors hit the market in the early 1970s, a computer that would have filled an entire room just a few years earlier suddenly fit on a single desktop. This marked the beginning of an unprecedented era of progress, with rapid advances in software development, computer architectures, and chip manufacturing technology.

Intel's cofounder Gordon Moore was among the first, in 1965, to observe a remarkable trend, now known as Moore's law: Every two years, the number of transistors per chip approximately doubles, and operating frequency increases, while power consumption and size stays about the same. This win‐win‐win situation was due to an intrinsic property of transistors: Smaller devices require less electric charge to switch on or off and can thus switch faster and dissipate less energy as heat when doing so.

Since proposed, Moore's law has held for more than half a century, bringing transistor counts from a humble 2300^6^ to 200 billion in a modern GPU^7^—an almost 90‐million‐fold increase!

Amid this digital revolution, Carver Mead, a professor at Caltech and himself a veteran of the field (he and Lynn Conway had literally just written the book on very‐large‐scale integration^8^), went against the common wisdom of the time and took another stab at analog computing.^9^

Mead realized that the same scaling laws, if applied to analog circuits, could finally enable the massive parallelism required for brain‐like systems. Intrigued by the remarkable resemblance between the subthreshold behavior of transistors and ion channels in the membrane of biological neurons, he and a small international community explored how to exploit these analog device characteristics to build much more compact and efficient models of neurons and sensors.

They adopted novel circuit design methodologies early on, for example, using floating‐gate transistors (now used in flash memory) as analog computing elements^10^ and pioneered event‐driven sensing with the silicon retina^11^ and cochleas.^12^ Their affinity for low‐level device physics, the emphasis on *efficiency* over raw performance, and the unorthodox approach became integral parts of the neuromorphic engineering creed.

But this artisanal style of circuit design (Carver Mead called it “eclectronics”^9^), ultimately struggled to keep pace with the rapid advances in synthesizable digital electronics. And since neural networks had fallen out of favor in artificial intelligence circles by the 1990s, there was little financial incentive to invest in a technology that could be upstaged by the next, twice‐as‐efficient generation of computers a mere two years later. Despite promising results, neuromorphic engineering thus remained a purely academic discipline.

## HARDWARE IN THE AGE OF DEEP LEARNING

**FIGURE 1:**
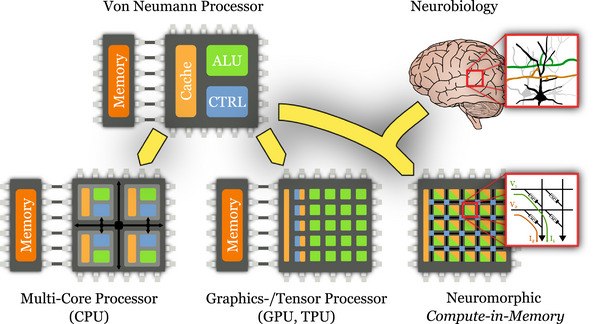
(Top left) The Von Neumann processor architecture separates computing elements (arithmetic logic units, ALUs, in green) and the finite‐state‐machines controlling them (CTRL, in blue) from on‐chip cache (light orange) and off‐chip memory (dark orange), which hold instructions and data. (Bottom left) Modern multicore processors (central processing units, CPUs) can perform multiple operations in parallel, each core autonomously executing a different program. (Bottom center) In graphics and tensor processing units (GPUs, TPUs), many more cores instead perform the same operations in lockstep on different parts of data stored in shared memory. (Top right) The brain does not separate computing elements and memory; instead, memory resides in the activation of neurons and the strengths of their synaptic connections. (Bottom right) Neuromorphic compute‐in‐memory architectures draw inspiration from both chip design and neuroscience. They combine processing and storage, for example, by storing synaptic connection strengths in the conductance of memristive nonvolatile memory devices (inset). Multiplication and addition are performed by scaling and summing the analog currents according to Ohm's and Kirchhoff's laws.

This situation changed drastically in 2011, when deep neural networks (DNNs^13^) made a roaring comeback by excelling at one image classification challenge after another.^14^, ^15^ These DNN models were not qualitatively different from their predecessors, but they were much *deeper*, that is, they had more layers and hence many more parameters. Training such large models had just become feasible, in part due to newly released public image data sets, and in part due to advances in computer hardware: By extending von Neumann's classic computer architecture to many cores that operate in parallel, multicore processors and graphics processing units (GPUs, see Figure 1) could finally deliver the necessary computing resources. Over the last decade, we have witnessed a Cambrian explosion of DNN models, topologies, and application areas—from image classification to large language models and generative artificial intelligence.

Meanwhile, parameter counts have grown from tens of millions^15^ to an astounding hundreds of billions^16^—that is an increase by five orders of magnitude in little more than a decade! Extrapolating another decade, the same rate of progress would finally bring us into the range of a thousand trillion parameters, exceeding the number of synaptic connections in the human brain.^17^ Many hope (or dread) that this would finally bring us within reach of human‐like artificial intelligence—the significance of which would be hard to overstate.

Perhaps they are right this time, and further incremental advancements will get us there, but I doubt it for three reasons.

First, current hardware platforms are ideally suited only for a specific class of DNNs that rely on the same operations from image processing that GPUs were designed for (i.e., dense matrix‐vector multiplications [MVMs] and convolutions): current platforms “won the hardware lottery,”^18^ so to speak. However, GPUs struggle with many other features that neuroscientists believe to be essential,^19^, ^20^, ^21^, ^22^ for example, the sparse and event‐driven activity of spiking neural networks (SNNs), sparse and irregular connectivity, low‐precision operations (up to sub‐bit precision), rich internal dynamics, flatter hierarchies with a high degree of recurrence, and plasticity.

Second, even Moore's law at its peak, that is, a doubling every 2 years, is too slow to keep up with current growth rates in AI—one 2018 study estimated a doubling every 3.4 months.^23^ So far, a hand‐full of companies have been able to make up for this discrepancy by using ever‐larger clusters of GPUs instead. For example, we know that Meta trained the recently published Llama 3.1 LLM on 16,000 powerful H100 GPUs,^24^ which cost around half a billion dollars. But such large systems come with a substantial communication overhead, resulting in less‐than‐linear increase in performance for a more‐than‐linear increase in hardware and energy cost.

Third, Moore's law itself is encroaching on absolute physical limits. At just a few nanometers across (for reference, a single silicon atom is around one‐fifth of a nanometer across), a modern transistor is now so small that electrons can randomly tunnel directly from one terminal to another, even when the transistor is supposed to be completely off. Rather than an ideal switch, the transistor thus behaves more and more like an unreliable, leaky valve. These leakage currents are now a major contributor to power consumption,^25^, ^26^ and shrinking transistors further will only exacerbate the problem. Clock frequencies have hit similar physical limits and have remained at the same level (around 1–2 GHz) since the early 2000s.^27^

With nowhere to go but up, semiconductor technology has started moving into the third dimension—first flipping the flat transistors on their side and then folding the gate around the channel. These devices, called FinFETs because they stand upright like fins, and their successors, called gate‐all‐around transistors, have smaller leakage and can thus be scaled down a bit further.^28^, ^29^ But these advances come with immense technical challenges and skyrocketing costs.

The natural next big step is truly three‐dimensional chips with multiple layers of active elements, but these face another fundamental limit: heat. Because all consumed power must be dissipated through the chip's surface area, adding more layers requires a proportional reduction in power consumption per layer—a trade‐off between area and activity that offers diminishing returns for conventional digital circuits.^27^, ^30^

All in all, it seems that “more Moore” alone will not save us this time. Instead, let us consider the alternatives that modern neuromorphic engineering has to offer.

## A NEUROMORPHIC FUTURE?

In a typical computer, most energy is not actually spent to perform computations *per se*, but rather to *communicate* data: from memory to cache, from cache to processors, or from one chip to another. For example, loading a byte from off‐chip memory takes a thousand times more energy than performing an addition.^31^

This is because every wire and transistor behaves like an unintended (or *parasitic*) capacitor, requiring energy to charge or discharge. The bigger this capacitance and the faster the clock, the more energy it consumes.

This is especially relevant for highly parallel systems like neural network accelerators, where many neurons need to communicate with each other in often unpredictable ways. And again, the brain provides inspiration for strategies to reduce the cost of communication, including access to memory.

First, rather than intentionally separating computing elements from memory, as is the case in von Neumann's computers, we can tightly interleave them—a concept known as *near‐memory* or *in‐memory* computing.^32^ Likewise, computing elements that frequently communicate with each other can be placed in closer proximity. This shortens the wires between them, obviates routing infrastructure, and thus reduces power.

Second, because the parameters of neural networks change slowly, if at all, they should remain stationary in memory. Emerging nonvolatile memory technologies, which retain their state even when the device is powered off, are particularly useful for this purpose as they require no additional energy to maintain, unlike conventional on‐chip memory.^33^ For instance, various types of memristor devices have been used to construct dense cross‐bar structures that store real‐valued parameters and perform highly efficient analog MVMs in memory, a crucial operation for DNNs.^34^, ^35^, ^36^ Figure 1 shows the working principle of a typical memristive compute‐in‐memory architecture.

Third, we should strive to avoid unnecessary communication by making each transmitted message as informative as possible, even if this comes at the expense of computation. The brain seems to abide by this principle, as evidenced by the sparse yet highly information‐dense code it uses to communicate sensory information. For example, neuroscientists estimate that retinal ganglion cells encode up to 7 bits of information into a single spike.^37^ This is possible, because the neuron is very precise about *when* it fires, hence the timing of a spike conveys a lot of information.

Finally, asynchronous and analog design techniques can drastically reduce power consumption, for example, by adiabatic switching,^38^, ^39^, ^40^ which can recover most of the spent energy, or by removing clock signals altogether, which can save a substantial amount of power.^41^

In sum, the path forward is clear: Larger, massively parallel systems of neurons (or populations thereof), arranged in dense three‐dimensional stacks, that communicate through sparse but informative messages and use local analog or mixed‐signal processing with tightly integrated (nonvolatile?) memory.

Many universities, start‐ups, and established companies have recently taken up some of these ideas and developed neuromorphic systems that range from event‐based sensors with impressive temporal resolution and dynamic range^42^, ^43^, ^44^ to low‐power AI accelerators,^45^, ^46^ to large‐scale systems for neuroscience research.^47^, ^48^, ^49^, ^50^

And yet—despite growing demand for AI and considerable effort by many over several decades—neuromorphic engineering has still rarely translated from academic research into commercially successful products. Why is that?

## A PATH FORWARD

I believe the biggest remaining challenge is cultural, rather than technical.

Following the physicist Richard Feynman's motto *there's plenty of room at the bottom*,^51^ the field has long pursued a bottom‐up approach, placing emphasis on elegant electronic circuits and devices that model certain low‐level properties of neurons, synapses, or learning rules. Fewer thoughts have been spared for the perhaps less exciting but equally important question of system architectures. Consequently, most neuromorphic research has focused on the niche of small and extremely low‐power edge devices, where system complexity is more manageable.

But this story undersells the true potential of neuromorphics. Reducing the power consumption of a small‐scale AI accelerator from milliwatts to microwatts may be an impressive technical achievement, but for all but a handful of niche applications, this is a drop in the bucket of total system power. Once we take into account the power consumption of the sensor that provides the data, the (wireless) communication interface, or even a simple status LED, a saved milliwatt is negligible. Instead, conventional chip design is finding more “room at the top”^52^: By simultaneously streamlining software, improving algorithms, and specializing hardware for different applications, chip capabilities continue to improve beyond Moore's law.

Meanwhile, research on SNN algorithms has struggled to hit a moving target by trying to directly replicate the successes of DNNs—algorithms which were specifically developed with GPUs in mind, and bear only a fleeting resemblance to what we now know about biological neural networks. The results have been mixed.^53^ Despite better alternatives,^54^ toy‐problems like handwritten digit recognition are still the most popular benchmarks for new neuromorphic chips—this does not make a strong case for the benefits of neuromorphic systems,^55^ and is hardly exciting for an audience used to deep learning on GPUs.

I think we need to aim higher. Power efficiency is a multiplicative factor and as such has the biggest leverage where the most power is used, where it is a substantial part of total system power, or where heat dissipation is a major concern. Rather than tiny ultra‐low power devices, the future of neuromorphic hardware might thus be in larger, very densely integrated three‐dimensional chips with power consumption in the (tens of) Watts range—just like the human brain.

This paradigm shift will not be easy, as it puts neuromorphic hardware in direct competition to GPUs. But if successful, it could open the doors to much more exciting applications, for example, in (autonomous) robotics, space applications, or even in data centers. And just as hardware has always shaped the development of AI, this might even facilitate a new type of “neuromorphic” intelligence that is more like our own.

## COMPETING INTERESTS

The author declares no competing interests.
